# Effect of Air Pollution Particulate Matter on Ischemic and Hemorrhagic Stroke: A Scoping Review

**DOI:** 10.7759/cureus.46694

**Published:** 2023-10-08

**Authors:** Krista Lamorie-Foote, Brandon Ge, Kristina Shkirkova, Qinghai Liu, William Mack

**Affiliations:** 1 Neurological Surgery, University of Southern California, Los Angeles, USA; 2 Neurological Surgery, Keck School of Medicine of University of Southern California, Los Angeles, USA

**Keywords:** hemorrhagic stroke, ischemic stroke, stroke, particulate matter, air pollution

## Abstract

Air pollution particulate matter (PM) exposure has been established as a risk factor for stroke. However, few studies have investigated the effects of PM exposure on stroke subtypes (ischemic and hemorrhagic stroke). Ischemic (IS) and hemorrhagic strokes (HS) involve distinctive pathophysiological pathways and may be differentially influenced by PM exposure. This review aims to characterize the effects of PM exposure on ischemic and hemorrhagic strokes. It also identifies subpopulations that may be uniquely vulnerable to PM toxicity. Pubmed was queried from 2000 to 2023 to identify clinical and epidemiological studies examining the association between PM exposure and stroke subtypes (ischemic and hemorrhagic stroke). Inclusion criteria were: 1) articles written in English 2) clinical and epidemiological studies 3) studies with a clear definition of stroke, IS, HS, and air pollution 4) studies reporting the effects of PM and 5) studies that included distinct analyses per stroke subtype. Two independent reviewers screened the literature for applicable studies. A total of 50 articles were included in this review. Overall, PM exposure increases ischemic stroke risk in both lightly and heavily polluted countries. The association between PM exposure and hemorrhagic stroke is variable and may be influenced by a country’s ambient air pollution levels. A stronger association between PM exposure and stroke is demonstrated in older individuals and those with pre-existing diabetes. There is no clear effect of sex or hypertension on PM-associated stroke risk. Current literature suggests PM exposure increases ischemic stroke risk, with an unclear effect on hemorrhagic stroke risk. Older patients and those with pre-existing diabetes may be the most vulnerable to PM toxicity. Future investigations are needed to characterize the influence of sex and hypertension on PM-associated stroke risk.

## Introduction and background

Stroke, a leading cause of morbidity and mortality, can be divided into two main subtypes: ischemic and hemorrhagic [1]. Ischemic stroke (IS) is characterized by decreased blood flow and tissue necrosis secondary to vascular obstruction [1]. Hemorrhagic stroke (HS) is defined by leakage of blood products into, or around, the brain via damaged blood vessels [1]. Stroke burden is expected to increase as the population ages [2]. It is therefore important to identify modifiable stroke risk factors. 

Ambient air pollution (AAP) is composed of particulate matter (PM), metals, and gaseous pollutants including ozone (O3), carbon monoxide (CO), sulphur dioxide (SO2), and nitrogen species (NO2, NOx) [3]. PM_2.5_ (<2.5µm) may be a significant contributor to AAP-associated toxicity given its small aerodynamic diameter and potential to enter into the systemic circulation after inhalation [3]. AAP exposure is a risk factor for stroke [3-7]. Air pollution can induce inflammation and oxidative stress, which may impact stroke incidence and/or progression [8]. While the association between PM and stroke is well characterized, few studies have investigated the differential effects of PM on IS and HS. The effects of PM may differ by stroke subtype, as IS and HS involve different pathways [1]. Patient-based factors may influence the effect of PM on stroke subtype. This review aims to examine the clinical association between PM and stroke subtype (IS and HS). Further, the review describes the influence of patient-based factors (age, comorbidities, sex) on the effects of PM exposure and stroke. 

## Review

Methods

Given the wide scope of this study, a scoping literature review was performed. PubMed was searched between 2000 and 2023 for relevant articles using keywords (Table 1). This review followed the Preferred Reporting Items for Systematic reviews and Meta-Analyses extension for Scoping Reviews (PRISM-ScR) guidelines. The initial search identified 1356 articles. Inclusion criteria were as follows: 1) clinical and epidemiological studies including humans 2) articles written in English 3) studies with a clear definition of stroke, IS, HS, and air pollution 4) studies that included separate analyses per stroke subtype 5) studies on the effects of PM. Studies on all other known air pollution constituents were excluded. 

**Table 1:** Search Criteria

Section	Search Terms
Ischemic Stroke	“particulate matter” or “air pollution” and “ischemic” and “stroke”
Hemorrhagic Stroke	“particulate matter” or “air pollution” and “hemorrhagic” and “stroke” “air pollution” and “ischemic” and “hemorrhagic” and “stroke”
Sex Differences	“air pollution” and “stroke” and “gender” and “sex”
Age Differences	“air pollution” and “stroke” and “age”
Comorbidities	“air pollution” and “stroke” and “hypertension” “air pollution” and “stroke” and “diabetes”

**Figure 1:**
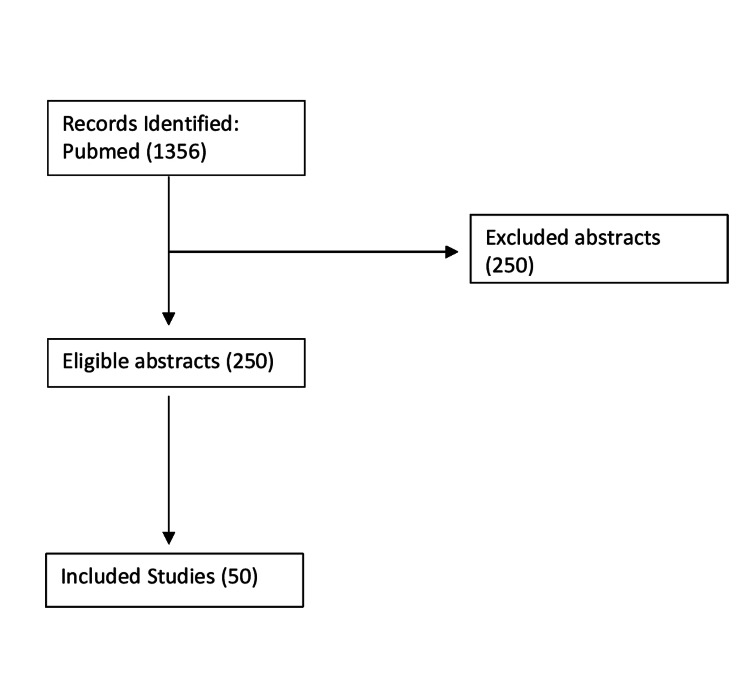
Flow Chart Outlining the Literature Search

Two independent reviewers performed the initial screen and reviewed the title and abstract of articles. The full text was read for articles that passed the initial screen. The initial screen included 250 articles. This review includes 50 articles (Figure 1). 

Ischemic stroke 

Short-Term Particulate Matter Exposure

An association between short-term PM exposure and IS has been established [6,9-26] (Table 2). Short-term PM exposure is defined as PM exposure for one to two days. A 2015 meta-analysis (Shah et al.) demonstrated that PM_2.5 _exposure was associated with a modest increase in combined IS admissions and deaths (Relative risk (RR): 1.010, 95% Cl: 1.008-1.011). PM_10_ (<10µm) showed a positive, but nonsignificant, association (RR: 1.002; 95% Cl: 0.999-1.994) with combined IS admissions and deaths [6]. The small size of PM_2.5_ may allow particles to cross into the systemic circulation, amplifying its neurotoxicity [3]. An earlier (2014) meta-analysis (Wang et al.) suggested a nonsignificant association between PM_10 _(RR: 1.0, 95% Cl: 0.976-1.024) and PM_2.5_ (RR: 1.013, 95% Cl: 0.958-1.070) on IS hospital admissions. A limited number of studies were included, which may have influenced the results [27]. 

**Table 2:** Short-term Particulate Matter Exposure and Ischemic Stroke *Results include short- and long-term PM exposure. Abbreviations: PM: particulate matter; IS: Ischemic stroke; HS: hemorrhagic stroke; ns: nonsignificant; UFP: ultrafine particle

Author, Year	Study	N	Location	Findings
Shah et al., 2015 [6]	Meta-analysis on PM and stroke-related hospital admission and mortality	20 studies on PM_2.5_ and IS, 19 studies on PM_10_ and IS	Worldwide	PM_2.5_ and PM_10_ significantly increased IS admission and mortality
Fu et al., 2019* [9]	Meta-analysis on PM_2.5 _and neurological disorders	23 studies on IS	Worldwide	PM_2.5_ significantly increased IS risk
Ban et al., 2021 [10]	Case-crossover study on PM­­_2.5_ and stroke incidence and mortality	107,604 IS cases, 7,342 IS deaths	China	PM_2.5_ significantly increased IS incidence and mortality
Hu et al., 2021 [11]	Time-series study on PM_2.5 _and IS hospital admissions	11,363 IS admissions	Yancheng, China	PM_2.5 _significantly increased same-day IS admissions
Tian et al., 2018 [12]	Time-series study on PM_2.5 _and IS hospital admissions	2,032,667 IS admissions	China	PM_2.5 _significantly increased same-day IS admissions
Zhang et al., 2018 [13]	Ecological study on PM_2.5_, PM_10 _exposure and stroke mortality	32,799 IS deaths	Beijing, China	PM_2.5_,PM_10_ significantly increased IS mortality
Dong et al., 2018 [14]	Time-series study on air pollutants and IS incidence and mortality	32,840 IS cases, 4,028 IS deaths	Changzhou, China	PM_10_ significantly increased IS mortality. PM_2.5 _increased IS mortality (ns)
Qian et al., 2013 [15]	Case-crossover study PM_10 _and stroke mortality in adults >65	30,583 IS deaths	Shanghai, China	PM_10_ significantly increased same-day IS mortality
Wang et al., 2020 [16]	Case-crossover study on air pollutants and IS incidence	63,997 IS cases	Shenzhen, China	High PM_10_ significantly increased IS incidence
Maheswaran et al., 2012 [17]	Ecological study on PM_10 _and HS, IS incidence	1,832 IS cases	London, UK	PM­_10_ increased IS incidence (ns)
Tian et al., 2017 [18]	Time-series study on PM_2.5_ and IS hospital admissions	63,956 IS admissions	Beijing, China	PM_2.5_ significantly increased same-day IS admissions
Wellenius et al., 2012 [19]	Case-crossover study on PM_2.5_ and IS incidence	1,705 IS cases	Boston, MA	Moderate PM_2.5_ significantly increased IS incidence.
Wellenius et al., 2005 [20]	Case-crossover study on PM_2.5 _and IS, HS hospital admissions among Medicare recipients	155,503 IS admissions	9 US cities	PM_10_ significantly increased same-day IS admissions
Yorifuji et al., 2011 [22]	Time-series study on PM_2.5_ and IS mortality	24,628 IS deaths	Tokyo, Japan	PM_2.5_ increased IS mortality (ns)
Oudin et al., 2010 [21]	Time-series and case-crossover study on PM_10_ and HS, IS admissions	11,267 IS admissions	Scania, Sweden	PM_10_ significantly increased IS admissions
Tian et al., 2019 [25]	Time-series study on PM_10-2.5_ and IS hospital admissions	2,032,667 IS admissions	China	PM_10-2.5_ significantly increased same-day IS admissions
Zhang et al., 2018 [26]	Case-crossover study on PM_2.5_ and cardiovascular hospital admissions	173,587 IS admissions	New York, USA	PM_2.5_ significantly increased IS admissions
Wing et al., 2015 [28]	Case-crossover study on PM_2.5_ and IS incidence	2,948 IS cases	Nueces County, Texas	PM_2.5_ not associated with IS incidence
Wang et al., 2014 [27]	Meta-analysis on PM_10_, PM_2.5_ and stroke admission	6 studies - PM_2.5_, 10 studies - PM_10_	Worldwide	PM_10 _not associated with IS admission. PM_2.5 _increased IS admission (ns)
Yang et al., 2014 [29]	Meta-analysis on air pollutants and stroke risk	8 studies - PM_2.5_, 21 studies - PM_10_	Worldwide	PM_2.5_, PM_10_ increased IS hospitalizations (ns)
Gu et al., 2020 [30]	Time-series study on PM_2.5_ and IS, HS hospital admissions	4,012,228 IS admissions	China	PM_2.5_ significantly increased same-day IS admissions
Andersen et al., 2010 [31]	Case-crossover study on ultrafine particles and stroke hospital admissions	6,798 IS admissions	Copenhagen, Denmark	UFP (particles <0.1um) significantly increased mild IS hospital admissions
Fisher et al., 2019 [32]	Case-crossover study on PM_2.5_, PM_10_ and IS, HS incidence in the Health Professionals Follow-up Study	539 IS cases	USA	PM_10_ significantly increased IS incidence
Mechtouff et al., 2012 [33]	Case-crossover study on air pollutants and IS incidence	376 IS cases	Lyon, France	PM_2.5_, PM_10_ not associated with IS incidence
Sun et al., 2019 [34]	Case-crossover study on air pollutants and IS, HS incidence in the Women’s Health Initiative	4,300 IS cases	USA	PM_2.5_, PM_10_ not associated with IS incidence
Butland et al., 2017 [35]	Case-crossover study on air pollutants and IS, HS incidence	1,311 IS cases	London, UK	PM_2.5_, PM_10_ not associated with IS incidence
Lin et al., 2016 [36]	Time-series study on PM and IS, HS mortality	5,113 IS deaths	Guangzhou, China	PM_10_, PM_2.5_, PM_1_ not associated with IS mortality
O’Donnell et al., 2011 [37]	Case-crossover study on PM_2.5_ and IS incidence	9,292 IS cases	Ontario, Canada	PM_2.5_ not associated with IS incidence
Sade et al., 2015 [23]	Case-crossover study on PM­_10_, PM_2.5 _and stroke admissions	4,325 IS cases	Israel	PM_10_, PM_2.5_ significantly increased same-day IS admissions
Huang et al., 2016 [24]	Case-crossover study on PM­_10_, PM_2.5_ and stroke admissions	130,774 IS cases	Beijing, China	PM_10_, PM_2.5_ significantly increased same-day IS admissions
Villeneuve et al., 2012 [38]	Case-crossover study on air pollutants and IS, HS hospital admissions	1,804 IS cases	Edmonton, Canada	PM_2.5_ not associated with IS admissions

A recent meta-analysis by Fu et al. (2019) demonstrated a stronger association between PM_2.5_ exposure and IS incidence than the meta-analyses conducted by Shah et al. and Wang et al. [6,9,27]. A greater proportion of studies were conducted in heavily polluted countries [9]. An established association between air pollution and stroke in heavily polluted countries may account for the more robust findings in the Fu et al. meta-analysis [6,9,29].

In heavily polluted countries, the association between short-term PM exposure and IS has been well documented [10-16,18,24,25,30]. Across China, a 10µg/m3 increase in PM_2.5_ increased IS incidence and mortality by 0.26% (95% Cl: 0.21-0.72%) and 1.09% (95% Cl: 0.05-2.14%), respectively. 43,300 stroke-related deaths and 48,800 incidences were attributable to PM_2.5_ exposure. PM_2.5_ was measured using county-level fixed monitoring data, which may bias the study’s estimates [10]. In Beijing, 10µg/m3 increases in PM_2.5_ and PM_10_ increased IS mortality by 0.23% (95% Cl: 0.04-0.42%) and 0.16% (95% Cl: 0.01-0.32%), respectively [13]. Across multiple studies, PM_2.5_ and PM_10_ exposure increased IS risk and mortality [11-16].

Moderate PM exposure may impact the IS risk. Moderate PM_2.5_ exposure (15-40µg/m^3^) increased IS risk by 34% when compared to light PM_2.5_ exposure (≤15µg/m^3^). PM_2.5_ levels were linearly associated with stroke risk and the association between PM_2.5_ and IS onset was strongest within 12 hours of exposure [19]. PM_2.5_ is neurotoxic at levels within the US National Ambient Air Quality Standards [39]. The effect of PM on IS may vary by stroke severity. Higher levels of ultrafine particle (UFP) (<0.1µm) exposure increased hospital admissions by 21% for mild IS without atrial fibrillation. There was no association between UFP exposure and severe IS admissions [31]. 

Other studies have found no association between PM exposure and IS [17,28,32-38]. The Health Professionals Follow-up Study (HPFS) demonstrated that daily changes in PM_10_, but not PM_2.5_, were associated with IS events. Subjects were mostly Caucasian men, and results, therefore, may not be generalizable to the entire population [32]. In the Women’s Health Initiative, no association was demonstrated between PM_2.5_/PM_10_ and IS risk [34]. Similarly, there was no association between PM_2.5_/PM_10_ and incident IS in the South London Stroke Register [35]. Exposure assessment, stroke ascertainment, and statistical approaches varied among studies, which could contribute to result heterogeneity. 

Long-Term Particulate Matter Exposure

Fewer studies have investigated the effects of long-term PM exposure on IS (Table 3). Long-term PM exposure is defined as PM exposure for one year or longer. A meta-analysis by Yuan et al. identified four studies that stratified analyses by stroke subtype [40]. Pooled results suggested no association between IS incidence and PM_2.5_ exposure (per 5µg/m^3^ increase, Hazard Ratio (HR): 1.62, 95% Cl: 0.88-2.97) [40-44]. In the Danish Nurse Cohort, IS risk increased by 17% (95% Cl: 1.01-1.34) per 5µg/m^3^ increase of one-year mean PM_2.5_. A 3µg/m^3^ increase of one-year mean PM_10_ showed a positive, but nonsignificant, association with IS risk (HR: 1.04, 95% Cl: 0.96-1.13). A linear dose-response relationship between PM_2.5_/PM_10_ and stroke incidence was noted. A threshold was found, above which an effect between PM and increased stroke risk was not observed (PM_2.5_, PM_10_: 20µg/m^3^) [45]. These results suggest that PM exposure can increase IS risk in a region with lower pollutant concentrations. Subjects were female nurses aged 44 years and older, limiting the study’s generalizability [45]. In contrast, PM_2.5_ exposure was not associated with IS incidence in the South London Stroke Register [46]. In Seoul, Korea, every 1µg/m^3^ increase in PM_2.5_/PM_10_ exposure increased IS incidence [42].The Atherosclerotic Cardiovascular Disease Risk in China (China-PAR) project demonstrated that every 10µg/m^3^ increase in PM_2.5_ increased IS incidence by 20% (95% Cl: 15-25%) [47]. An almost linear association between PM_2.5_ exposure and incident IS was noted [47]. Higher air pollution levels in Korea and China relative to Western countries may account for the increased risks of PM exposure [29]. 

**Table 3:** Long-Term Particulate Matter Exposure and Ischemic Stroke Abbreviations: PM: particulate matter; IS: Ischemic stroke; HS: hemorrhagic stroke; ns: nonsignificant; DALYs: disability-associated life years

Author, Year	Methods	N	Location	Findings
Yuan et al., 2019 [40]	Meta-analysis of cohort studies on PM_2.5 _and stroke incidence	4 studies on IS	Worldwide	PM_2.5_ increased IS incidence (ns)
Cai et al., 2018 [44]	Population-based study on air pollutants and cardiovascular disease	923 IS cases	Norway and UK	PM_2.5 _increased IS incidence (ns)
Qiu et al., 2017 [41]	Cohort study on PM_2.5_ and stroke admission in adults ≥65 years	3,526 IS cases	Hong Kong, China	PM_2.5_ significantly increased IS incidence
Kim et al., 2017 [42]	Population-based study on PM_2.5_ and cardiovascular events	688 IS cases	Seoul, Korea	PM_2.5_, PM_2.5-10 _significantly increased IS incidence
Puett et al., 2011 [43]	Cohort study on PM_2.5_, PM_10_, and cardiovascular disease	230 IS cases	USA	PM_2.5_, PM_10_not associated with IS risk
Amini et al., 2020 [45]	Danish Nurse Cohort study on PM_2.5_, PM_10_, and stroke incidence	944 IS cases	Denmark	One-year PM_2.5_ significantly increased IS incidence, one-year PM_10_ increased IS incidence (ns)
Crichton et al., 2016 [46]	South London Stroke Register study on PM_2.5_, PM_10_, and stroke incidence	1,350 IS cases	London, UK	PM_2.5_, PM_10_ not associated with IS incidence
Huang et al., 2019 [47]	Prospective cohort study on PM_2.5 _exposure and stroke incidence	2,230 IS cases	Beijing, China	PM_2.5_ significantly increased IS incidence
Maheswaran et al., 2010 [48]	Population-based study on air pollutants and poststroke survival	1,856 poststroke deaths	London, UK	Poststroke PM­_10_ not associated with cerebral infarction mortality
Chen et al., 2019 [49]	China National Stroke Registry cohort study on prestroke PM_1_, PM_2.5_, PM_10_, and fatal IS incidence	12,291 IS patients, 1,649 IS deaths	China	PM_1_ and PM_2.5_ significantly increased incident fatal IS. PM_10_ not associated with fatal IS
Jiang et al., 2020 [5]	Global Burden of Disease Study 2017 on PM_2.5_-attributable stroke burden	10,515,500 stroke DALYS, 444,940 stroke deaths attributable to PM_2.5_	Worldwide	3,950,200 IS DALYS and 183,523 IS deaths attributable to PM_2.5_ in 2017

Long-term PM exposure may impact survival after IS. Three-year prestroke PM_1_ (≤1µm) and PM_2.5_ exposure increased mortality in the year following IS (PM_1_: HR 1.05, 95% Cl: 1.02-109; PM_2.5_: HR 1.03, 95% Cl: 1.00-1.06) across China [49]. Maheswaran et al. found no association between poststroke PM_10_ exposure and increased mortality after cerebral infarction in South London (HR: 1.3, 95% Cl: 0.84-2.01) [48]. The same group later demonstrated no effect of long-term residential PM_10_ exposure on incident IS [17]. 

Hemorrhagic stroke 

Research examining PM and HS is limited and has produced inconsistent results [6,9,10,13,15,17,20-24,30,32,34-36,38,42,45-47,50,51] (Table 4). 6,565,200 million HS-related DALYs were attributable to PM_2.5_ in 2017 [5]. Pooled analyses from Shah et al. demonstrated no association between short-termPM_2.5_ (RR: 1.004, 95% Cl: 0.978-1.029) or PM_10_ exposure (1.002, 95% Cl: 0.997-1.006) and combined HS admission and mortality [6]. PM_2.5_ was (nonsignificantly) associated with HS incidence in Fu et al. (RR: 1.04, 95% Cl: 1.0-1.07) [9]. 

**Table 4:** Short- and Long-Term Particulate Matter Exposure and Hemorrhagic Stroke *After adjustment for nitrogen oxides (NOx). †Results include short- and long-term PM exposure. Abbreviations: PM: particulate matter; IS: ischemic stroke; HS: hemorrhagic stroke; SAH: subarachnoid hemorrhage; ns: nonsignificant; ICH: intracerebral hemorrhage; DALYs: disability-associated life years

Author, Year	Methods	N	Location	Findings	Stronger association between PM and HS or IS?
Short-term PM exposure					
Shah et al., 2015 [6]	Meta-analysis on PM and stroke-related hospital admission and mortality	5 studies - PM_2.5_, 12 studies - PM_10_	Worldwide	PM_2.5_, PM_10_ not associated with HS admission and mortality	IS
Ban et al., 2021 [10]	Case-crossover study on PM­­_2.5_ and stroke incidence and mortality	19,100 HS cases, 11,922 HS deaths	China	PM_2.5_ not associated with HS incidence and mortality	IS
Cai et al., 2020 [51]	Case-crossover study on PM­­_2.5 _and fatal HS incidence	6,412 fatal HS cases	Shanghai, China	PM_2.5 _significantly increased fatal HS	N/A
Zhang et al., 2018 [13]	Ecological study on PM_2.5_, PM_10_ exposure and stroke mortality	13,051 HS deaths	Beijing, China	PM_2.5_ significantly increased HS mortality	PM_2.5_:HS, PM_10_: IS
Maheswaran et al., 2012 [17]	Ecological study on PM_10 _and HS, IS incidence	348 HS cases	London, UK	PM­_10_ not associated with HS incidence	N/A
Wellenius et al., 2005 [20]	Case-crossover study on PM_2.5 _and IS, HS hospital admissions among Medicare recipients	19,314 HS admissions	9 US cities	PM_10_ not associated with HS admissions	IS
Oudin et al., 2010 [21]	Time-series and case-crossover study on PM_10_ and HS, IS admissions	1,681 HS admissions	Scania, Sweden	PM_10_ not associated with HS admissions	IS
Gu et al., 2020 [30]	Time-series study on PM_2.5_ and IS, HS hospital admissions	1,089,239 HS admissions	China	PM_2.5_ negatively associated with HS admissions (ns)	IS
Fisher et al., 2019 [32]	Case-crossover study on PM_2.5_, PM_10_ and IS, HS incidence in the Health Professionals Follow-up Study	122 HS cases	USA	PM_2.5_, PM_10 _not associated with HS incidence	PM_10_: IS
Sun et al., 2019 [34]	Case-crossover study on air pollutants and IS, HS incidence in the Women’s Health Initiative	924 HS cases	USA	PM_10_, PM_2.5_ not associated with HS incidence	N/A
Butland et al,. 2017 [35]	Case-crossover study on air pollutants and IS, HS incidence	256 HS cases	London, UK	PM_2.5_, PM_10_ significantly negatively associated with HS incidence^*^	N/A
Lin et al., 2016 [36]	Time-series study on PM and IS, HS mortality	3,953 HS deaths	Guangzhou, China	PM_10_, PM_2.5_, PM_1_ significantly increased HS mortality	HS
Villeneuve et al., 2012 [38]	Case-crossover study on air pollutants and IS, HS hospital admissions	909 HS cases	Edmonton, Canada	PM_2.5_ not associated with HS admissions	N/A
Sade et al., 2015 [23]	Case-crossover study on PM­_10_, PM_2.5_, and stroke admissions	512 HS cases	Israel	PM_10_, PM_2.5_ not associated with HS admissions	IS
Huang et al., 2016 [24]	Case-crossover study on PM­_10_, PM_2.5_, and stroke admissions	16,880 HS cases	Beijing, China	PM_10_, PM_2.5_ associated with HS on warm days	IS
Qian et al., 2019 [50]	Case-crossover study on PM_2.5_ and fatal ICH incidence	5,286 fatal ICH cases	Shanghai, China	PM_2.5_ significantly increased fatal ICH incidence	N/A
Qian et al., 2013 [15]	Case-crossover study PM_10 _and stroke mortality in adults >65 years	17,582 HS deaths	Shanghai, China	PM_10_ not associated with HS mortality	IS
Yorifuji et al., 2011 [22]	Time-series study on PM_2.5_ and IS mortality	4,983 SAH deaths 11,829 ICH deaths	Tokyo, Japan	PM_2.5_ increased ICH mortality (ns). PM_2.5 _increased SAH mortality.	HS (SAH)
Fu et al., 2019^† ^[9]	Meta-analysis on PM_2.5 _and neurological disorders	13 studies on HS	Worldwide	PM_2.5_ increased HS risk (ns)	HS
Long-term PM exposure					
Yuan et al., 2019 [40]	Meta-analysis of cohort studies on PM_2.5_ and stroke incidence	4 studies on HS	Worldwide	PM_2.5_ increased HS incidence (ns)	IS
Crichton et al., 2016 [46]	South London Stroke Register study on PM_2.5_, PM_10_, and stroke incidence	450 HS cases	London, UK	PM_2.5_, PM_10_ not associated with HS incidence	N/A
Huang et al., 2019 [47]	Prospective cohort study on PM_2.5_ exposure and stroke incidence	973 HS cases	Beijing, China	PM_2.5_ significantly increased HS incidence	IS
Jiang et al., 2020 [5]	Global Burden of Disease Study 2017 on PM_2.5_-attributable stroke burden	10,515,500 stroke DALYS, 444,940 stroke deaths attributable to PM_2.5_	Worldwide	3,277,200 HS DALYS and 261,417 HS deaths attributable to PM_2.5_ in 2017	N/A
Puett et al., 2011 [43]	Cohort study on PM_2.5_, PM_10_,and cardiovascular disease	70 HS cases	USA	PM_2.5_, PM_10 _not associated with HS risk	N/A
Qiu et al., 2017 [41]	Cohort study on PM_2.5_ and stroke admission in adults ≥65 years	1,175 HS cases	Hong Kong, China	PM_2.5 _not associated with HS incidence	IS
Kim et al., 2017 [42]	Population-based study on PM_2.5_ and cardiovascular events	292 HS cases	Seoul, Korea	PM_2.5_, PM_2.5-10 _significantly increased HS incidence	PM_2.5_: IS, PM_2.5-10_:HS
Amini et al., 2020 [45]	Danish Nurse Cohort study on PM_2.5_, PM_10_, and stroke incidence	134 HS cases	Denmark	One-year PM_2.5_, PM­_10 _increased HS incidence (ns)	IS

An association between short-term PM_2.5_/PM_10_ exposure and HS incidence was observed in the HPFS study on the day before stroke [32]. This association was not consistent across exposure days [32]. Gu et al. noted similar results, with an association between PM_2.5_ and HS hospital admissions that was significant with specific single-day exposures or moving average exposures [30]. Interestingly, same-day PM_10_ exposure decreased HS incidence in London after adjusting for NO_x_. As PM_2.5_ and NO_x_ are strongly correlated, this association may be secondary to collinearity [35]. 

No association between one- or three-year mean PM_2.5_ or PM_10_ and incident HS was demonstrated in the Danish Nurse Cohort [45]. Similarly, no association between daily PM_2.5_/PM_10_ exposure and incident HS was noted in the Women’s Health Initiative [34]. In contrast, Kim et al. demonstrated that every 1µg/m^3^ increase in long-term PM_2.5_ and PM_10_ concentration increased HS incidence [42]. In the China-PAR project, every 10µg/m^3^ increase in long-term PM_2.5_ increased incident HS by 12% (95% Cl: 5-20%) [47].

Short-term PM_2.5_ increased HS (excess risk (ER): 14% (95% Cl: 2-27%)) but not IS mortality (3%, 95% Cl: -6-14%) in Guangzhou, China. Stroke mortality rate in the study region was lower than in other industrialized cities in China, suggesting data underreporting [36]. Similarly, elevated PM_2.5_ levels two days before stroke increased fatal intracerebral hemorrhagic stroke (ICH) in Shanghai, China [50]. In Beijing, China, a 10µg/m^3^ increase in same-day PM_2.5_ increased HS mortality by 0.37% (95% Cl: 0.07-0.67%) [13]. 

The majority of studies report no association between PM and HS [10,15,17,20,23,24,34,38,45,46]. North American and European studies tend to report no association between PM and HS, while studies from Asia suggest an association [13,17,20,21,36,38,42,45-47,51]. As pollution exposure concentrations tend to be lower in Western countries, the average ambient pollution concentration may influence the effect of PM on HS [29].

Comparison of particulate matter effects on ischemic and hemorrhagic stroke 

The association between PM exposure and IS is well established, while the association between PM exposure and HS is less clear. Most studies reported stronger associations of PM with IS than HS, while few reported the opposite effect [6,9,10,13,21,23,36,42,45,47,52,53] (Table 4). PM_10_ levels were associated with same-day IS, but not HS, admissions among Medicare recipients [20]. In China, every 10µg/m^3^ increase in PM_2.5_ concentration, increased years life lost by 0.31% (95% Cl: 0.15-0.46) for IS and 0.23% (95% Cl: 0.09-0.36) for HS [53]. The differential effect of PM on stroke subtypes may be secondary to the distinctive underlying mechanisms.

While lifestyle factors, such as alcohol use and smoking, are risk factors for IS and HS, these factors may be more strongly associated with HS [54,55]. Current smoking was more strongly associated with HS (HR 2.56, 95% Cl: 1.92-3.41) than IS risk (1.62, 95% Cl: 1.39-1.90) in a cohort study [56]. Heavy alcohol consumption increased HS (2.73, 95% Cl: 1.83-4.07) but not IS risk (1.10, 95% Cl: 0.83-1.47) [56]. However, opposite trends have also been noted [57]. Risk factors demonstrating greater association with IS than HS include pre-existing diabetes, previous myocardial infarction, previous stroke, and atrial fibrillation [54,58]. Air pollution exposure has been proposed as a risk factor for cardiovascular disease and diabetes [3,59]. 

Pathophysiology behind air pollution-associated stroke 

**Figure 2:**
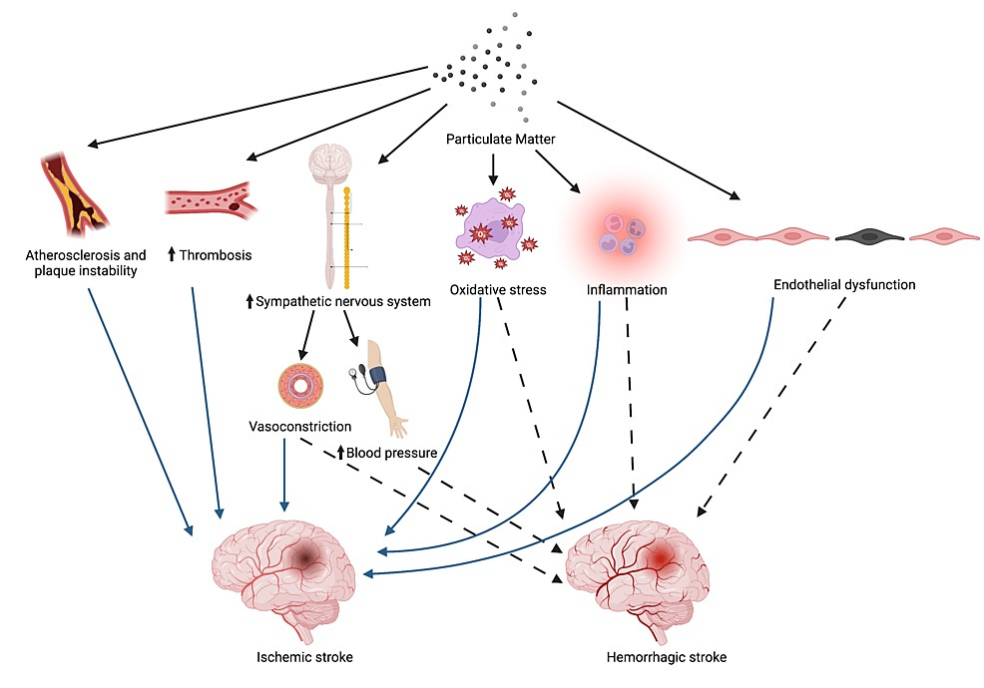
Mechanisms Underlying Particulate Matter Exposure and Ischemic and Hemorrhagic Stroke Figure created with BioRender.com

The exact mechanisms underlying air pollution-associated ischemic and hemorrhagic stroke are still unknown. PM exposure activates neuroinflammatory and oxidative stress pathways in both clinical and animal studies [8,60-63]. In addition, PM exposure contributes to endothelial dysfunction, which is a risk factor for stroke [7,62,64]. Atherosclerosis is an important mechanism underlying IS development [65]. PM exposure contributes to the formation and progression of atherosclerosis [62]. Furthermore, PM exposure is associated with increased plaque instability [62]. PM exposure increases sympathetic nervous system activity [66]. These effects may cause increases in blood pressure and risk of thrombosis [67]. Thrombus formation may contribute to IS pathogenesis, while hypertension increases HS and IS risk [58]. Proposed mechanisms behind air pollution particulate matter and ischemic and hemorrhagic stroke have been outlined in Figure 2. 

Effect of particulate matter on stroke in susceptible populations 

The effect of PM exposure on IS and HS may depend on patient-based factors, such as sex, age, and comorbidities. This study investigated the influence of sex, age, and comorbidities (hypertension and diabetes) on the association between PM exposure and stroke. 

Sex

Studies have produced inconsistent results with regards to the influence of sex on PM exposure and stroke risk (Table 5). PM_2.5_ exposure (nonsignificantly) increased stroke incidence and mortality in females compared to males across China. Chinese females have a higher prevalence of diabetes and hypertension, which may render them vulnerable to PM neurotoxicity [10,68]. In contrast, the effect of PM_10_ on daily IS deaths was stronger in males than females in Changzhou, China. Study authors suggested that males may spend more time outdoors and be exposed to higher PM_10 _levels [14]. Globally, males had a higher burden of PM-attributable stroke (stroke-related DALYs and deaths) in 2017 [5]. Risk factors for stroke, such as smoking, tend to be more prevalent in men [69]. 

**Table 5:** Influence of Sex on Particulate Matter Exposure and Stroke *No sex differences indicate that sex differences were examined in each study, and sex did not influence the association between particulate matter exposure and stroke Abbreviations: PM: particulate matter; IS: ischemic stroke; HS: hemorrhagic stroke

Author, Year	Findings
Ban et al., 2021 [10]	PM_2.5 _stronger association with stroke incidence and mortality in females
Hu et al., 2021 [11]	PM_2.5_ stronger association with IS admissions in females
Tian et al., 2018 [12]	No sex differences
Zhang et al., 2018 [13]	No sex differences
Dong et al., 2018 [14]	PM_10_ stronger association with IS mortality in males
Qian et al., 2013 [15]	PM_10 _stronger association with stroke and HS mortality in males
Tian et al., 2017 [18]	No sex differences
Gu et al., 2020 [30]	No sex differences
Qiu et al., 2017 [41]	PM_2.5 _stronger association with IS admission in males
Chen et al., 2019 [49]	PM_1 _stronger association with fatal stroke in males
Qian et al., 2019 [45]	No sex differences
Jiang et al., 2020 [5]	Higher burden of PM_2.5_-attributable stroke in males

Age

Older individuals are susceptible to air pollution toxicity [70]. Likewise, a stronger association between PM and stroke may be observed in older populations (Table 6). Chinese adults aged ≥75 years had an increased stroke mortality risk after PM_2.5_ exposure compared to individuals <75 years. However, the association between PM_2.5_ and stroke incidence increased in adults aged <75 years compared to adults ≥75 years [10]. The average age for first-ever stroke ranges from 60.9 to 63.4 years in China [68]. Therefore, the effect of PM_2.5_ on stroke incidence may be greatest in individuals aged ≥60 years and not ≥75 years. Similarly, the association between PM_2.5_ and IS admissions was increased (nonsignificantly) in Chinese adults aged 65-74 compared to adults aged <65 [30]. A greater effect of PM_2.5_ on IS admissions was demonstrated in adults ≥75 years in Yancheng, China [11]. For HS, PM_2.5_ exposure increased fatal ICH incidence among Chinese adults >65 years but not in adults <65 years [50]. It is possible that individuals aged ≥60 years are more vulnerable to PM toxicity [10]. 

**Table 6:** Influence of Age on Particulate Matter Exposure and Stroke *No effect indicates that age was examined in each study and did not affect the association between particulate matter exposure and stroke Abbreviations: PM: particulate matter; IS: ischemic stroke; HS: hemorrhagic stroke; ICH: intracerebral hemorrhage; yrs: years

Author, Year	Findings
Ban et al., 2021 [10]	PM_2.5_ stronger association with stroke mortality in people ≥75 yrs and stroke incidence in people 64-74 yrs
Hu et al., 2021 [11]	PM_2.5_ stronger association with IS admissions in people ≥75 yrs
Tian et al., 2018 [12]	PM_2.5_ stronger association with IS admissions in people ≥75 yrs
Zhang et al., 2018 [13]	No effect
Dong et al., 2018 [14]	PM_10_, PM_2.5_ stronger association with IS mortality in people ≤65 yrs
Qian et al., 2013 [15]	PM_10_ stronger association with stroke mortality in people 65-75 yrs
Wang et al., 2020 [16]	High PM_10_ exposure stronger association with IS incidence in people ≥65 yrs
Maheswaran et al., 2012 [17]	PM_10_ stronger association with IS incidence in people 65-79 yrs
Wellenius et al., 2012 [19]	No effect
Gu et al., 2020 [30]	PM_2.5_ stronger association with IS admissions in people 65-74 yrs
Fisher et al., 2019 [32]	No effect
Huang et al., 2019 [47]	PM_2.5_ stronger association with IS incidence in people ≥60 yrs
Chen et al., 2019 [49]	PM_1 _stronger association with fatal IS in people ≥75 yrs
Sade et al., 2015 [23]	PM_10_, PM_2.5_ stronger association with IS admissions in people <55 yrs
Huang et al., 2016 [24]	No effect
Qian et al., 2019 [45]	PM_2.5_ stronger association with fatal ICH in people ≥65 yrs

In London, the strongest IS risk per 10µg/m^3^ increase in PM_10_ occurred in adults aged 65-79 years (rate ratio 1.96, 95% Cl: 1.10-3.13) compared to adults aged 45-64 years (1.12, 95% Cl: 0.55-2.28) and adults older than 80 (0.5, 95% Cl: 0.19-1.32). The data used for census counts of individuals >80 years may have been inaccurate in the study’s small region. This may contribute to the lack of association between PM_10_ and IS in adults >80 years [17]. There was no association between PM_10_ exposure and HS incidence across age ranges [17]. 

Several studies have reported no effect of age on PM and stroke [13,32]. In Changzhou, China, PM_10_-related IS mortality risk was greater in individuals <65 years compared to individuals ≥65 years. Young individuals may spend more time outdoors and be exposed to higher PM_10_ levels than elderly individuals [14]. 

Hypertension and Diabetes

Individuals with underlying comorbidities, such as diabetes and hypertension, may be vulnerable to the adverse effects of PM (Table 7). PM_2.5_ exposure increased IS risk by 10.6% among patients with diabetes (95% Cl: 0.8-21.5%) in Canada, a region with low air pollution levels. No association was demonstrated between PM_2.5_ and IS among patients without diabetes [37]. PM_2.5_ exposure increased fatal ICH by 26% (95% Cl: 9-46%) among patients with diabetes in Shanghai. This association was significantly stronger when compared to subjects without diabetes (5% increase, 95% Cl: -2-12%) [50]. The exact mechanisms behind why patients with diabetes may be more vulnerable to PM exposure are still unknown; however, inflammation may play a key role. Diabetes can increase vascular inflammation and cause endothelial dysfunction, which may contribute to an individual’s susceptibility to PM_2.5_ neurotoxicity [71]. 

**Table 7:** Effect Modification of Hypertension and Diabetes on Particulate Matter Exposure and Stroke *No effect means that these factors were examined in each study and there was no effect of hypertension or diabetes on stroke Abbreviations: PM: particulate matter; IS: ischemic stroke; HS: hemorrhagic stroke; ICH: intracerebral hemorrhage

Author, Year	Findings
Qian et al., 2013 [15]	No effect of hypertension or diabetes
Wellenius et al., 2012 [19]	No effect of hypertension or diabetes
Fisher et al., 2019 [32]	No effect of hypertension or diabetes
Sun et al., 2019 [34]	No effect of hypertension or diabetes
O’Donnell et al., 2011 [37]	PM_2.5_ stronger association with IS incidence in people with diabetes. No effect of hypertension
Huang et al., 2019 [47]	No effect of hypertension or diabetes
Qian et al., 2019 [45]	PM_2.5 _stronger association with fatal ICH in people with diabetes. No effect of hypertension.
Cai et al., 2020 [51]	PM_2.5_ stronger association with fatal HS in people with hypertension

A similar effect was not seen among patients with hypertension. While patients with hypertension had an 8% increase (95% Cl: 0-16%) in fatal ICH incidence after greater PM_2.5_ exposure, this association was not significant [50]. Similarly, there was no effect of hypertension on PM_2.5_ and IS [37]. In contrast, PM_2.5_ exposure increased fatal HS incidence by 6% (95% Cl: 2-10%) in patients with hypertension but did not increase fatal HS in patients without hypertension (4%, 95% Cl: -2-11) [51]. PM_2.5_ exposure can activate the sympathetic nervous system and induce vasoconstriction, which may further increase the blood pressure of patients with hypertension and cause hemorrhage [62,66]. 

Multiple studies have reported no effect of diabetes or hypertension on stroke risk after PM_2.5_ exposure [19,32,34,47]. In the Women’s Health Initiative, neither pre-existing diabetes nor hypertension increased PM_2.5_ and PM_10_-attributable stroke risk [34]. Post-menopausal women were analyzed, limiting the study’s generalizability. The China-PAR project found no effect of diabetes or hypertension on PM_2.5_-related stroke, IS, or HS risk [47]. Studies included in this section are from both high-income and developing countries. Overall, the data is mixed with some studies suggesting an effect of hypertension and diabetes on stroke, while others do not. There is no clear difference between high-income and developing countries, but there are too few studies to make definitive conclusions. 

Limitations and next steps 

A limitation of this review is that PubMed was the sole database that was queried and therefore studies that were present in other databases may have been missed. Our PubMed search resulted in a wide range of studies and it was felt that the literature was adequately summarized through our search. There have been a number of prior literature reviews focusing on the association between air pollution and stroke. However, this review investigates the effects of both short- and long-term PM exposure on IS and HS, as well as identifies possible factors that may influence the association between PM and stroke. 

The majority of the studies used were retrospective (19 case-crossover, eight time-series, two ecological studies, five database studies). There were five prospective cohort studies. A total of five meta-analyses were included in this review. Prospective studies are subject to less bias than retrospective studies, which may have influenced our results given that a majority of the studies were retrospective in nature [72].

Overall, data on the association between particulate matter and hemorrhagic stroke is limited and requires additional research. Future studies are needed to clearly delineate whether sex influences the association between PM and stroke. There have been a limited number of studies evaluating the effects of hypertension and other comorbidities on PM-associated stroke. Future investigations should focus on the influence of comorbidities on PM-associated ischemic and hemorrhagic stroke.

## Conclusions

Particulate matter exposure differentially influences ischemic and hemorrhagic stroke risk. Short- and long-term PM exposure increases IS risk in heavily and lightly polluted regions. The association between PM exposure and HS risk is less clear and may depend on the level of ambient air pollution present in a country. Older patients and patients with pre-existing diabetes may be uniquely susceptible to the adverse effects of PM. Future studies are needed to determine the effect of PM exposure on HS and the influence of sex and hypertension on PM-associated stroke risk. Improving air quality standards and monitoring those most vulnerable to PM toxicity may mitigate the detrimental health effects of PM and reduce healthcare costs. 

## References

[1] Kuriakose D, Xiao Z (2020). Pathophysiology and treatment of stroke: present status and future perspectives. Int J Mol Sci.

[2] Béjot Y, Bailly H, Graber M (2019). Impact of the ageing population on the burden of stroke: the Dijon Stroke Registry. Neuroepidemiology.

[3] Al-Kindi SG, Brook RD, Biswal S, Rajagopalan S (2020). Environmental determinants of cardiovascular disease: lessons learned from air pollution. Nat Rev Cardiol.

[4] Babadjouni RM, Hodis DM, Radwanski R, Durazo R, Patel A, Liu Q, Mack WJ (2017). Clinical effects of air pollution on the central nervous system; a review. J Clin Neurosci.

[5] Jiang Y, Lu H, Man Q (2020). Stroke burden and mortality attributable to ambient fine particulate matter pollution in 195 countries and territories and trend analysis from 1990 to 2017. Environ Res.

[6] Shah AS, Lee KK, McAllister DA (2015). Short term exposure to air pollution and stroke: systematic review and meta-analysis. BMJ.

[7] Kulick ER, Kaufman JD, Sack C (2023). Ambient air pollution and stroke: an updated review. Stroke.

[8] Liu Q, Shkirkova K, Lamorie-Foote K (2021). Air pollution particulate matter exposure and chronic cerebral hypoperfusion and measures of white matter injury in a murine model. Environ Health Perspect.

[9] Fu P, Guo X, Cheung FM, Yung KK (2019). The association between PM(2.5) exposure and neurological disorders: a systematic review and meta-analysis. Sci Total Environ.

[10] Ban J, Wang Q, Ma R (2021). Associations between short-term exposure to PM(2.5) and stroke incidence and mortality in China: a case-crossover study and estimation of the burden. Environ Pollut.

[11] Hu W, Chen Y, Chen J (2021). Short-term effect of fine particular matter on daily hospitalizations for ischemic stroke: a time-series study in Yancheng, China. Ecotoxicol Environ Saf.

[12] Tian Y, Liu H, Zhao Z (2018). Association between ambient air pollution and daily hospital admissions for ischemic stroke: a nationwide time-series analysis. PLoS Med.

[13] Zhang R, Liu G, Jiang Y (2018). Acute effects of particulate air pollution on ischemic stroke and hemorrhagic stroke mortality. Front Neurol.

[14] Dong H, Yu Y, Yao S (2018). Acute effects of air pollution on ischaemic stroke onset and deaths: a time-series study in Changzhou, China. BMJ Open.

[15] Qian Y, Zhu M, Cai B (2013). Epidemiological evidence on association between ambient air pollution and stroke mortality. J Epidemiol Community Health.

[16] Wang Z, Peng J, Liu P (2020). Association between short-term exposure to air pollution and ischemic stroke onset: a time-stratified case-crossover analysis using a distributed lag nonlinear model in Shenzhen, China. Environ Health.

[17] Maheswaran R, Pearson T, Smeeton NC, Beevers SD, Campbell MJ, Wolfe CD (2012). Outdoor air pollution and incidence of ischemic and hemorrhagic stroke: a small-area level ecological study. Stroke.

[18] Tian Y, Xiang X, Wu Y (2017). Fine particulate air pollution and first hospital admissions for ischemic stroke in Beijing, China. Sci Rep.

[19] Wellenius GA, Burger MR, Coull BA (2012). Ambient air pollution and the risk of acute ischemic stroke. Arch Intern Med.

[20] Wellenius GA, Schwartz J, Mittleman MA (2005). Air pollution and hospital admissions for ischemic and hemorrhagic stroke among medicare beneficiaries. Stroke.

[21] Oudin A, Strömberg U, Jakobsson K, Stroh E, Björk J (2010). Estimation of short-term effects of air pollution on stroke hospital admissions in southern Sweden. Neuroepidemiology.

[22] Yorifuji T, Kawachi I, Sakamoto T, Doi H (2011). Associations of outdoor air pollution with hemorrhagic stroke mortality. J Occup Environ Med.

[23] Yitshak Sade M, Novack V, Ifergane G, Horev A, Kloog I (2015). Air pollution and ischemic stroke among young adults. Stroke.

[24] Huang F, Luo Y, Guo Y (2016). Particulate matter and hospital admissions for stroke in Beijing, China: modification effects by ambient temperature. J Am Heart Assoc.

[25] Tian Y, Liu H, Xiang X (2019). Ambient coarse particulate matter and hospital admissions for ischemic stroke. Stroke.

[26] Zhang W, Lin S, Hopke PK (2018). Triggering of cardiovascular hospital admissions by fine particle concentrations in New York state: before, during, and after implementation of multiple environmental policies and a recession. Environ Pollut.

[27] Wang Y, Eliot MN, Wellenius GA (2014). Short-term changes in ambient particulate matter and risk of stroke: a systematic review and meta-analysis. J Am Heart Assoc.

[28] Wing JJ, Adar SD, Sánchez BN, Morgenstern LB, Smith MA, Lisabeth LD (2015). Ethnic differences in ambient air pollution and risk of acute ischemic stroke. Environ Res.

[29] Yang WS, Wang X, Deng Q, Fan WY, Wang WY (2014). An evidence-based appraisal of global association between air pollution and risk of stroke. Int J Cardiol.

[30] Gu J, Shi Y, Chen N, Wang H, Chen T (2020). Ambient fine particulate matter and hospital admissions for ischemic and hemorrhagic strokes and transient ischemic attack in 248 Chinese cities. Sci Total Environ.

[31] Andersen ZJ, Olsen TS, Andersen KK, Loft S, Ketzel M, Raaschou-Nielsen O (2010). Association between short-term exposure to ultrafine particles and hospital admissions for stroke in Copenhagen, Denmark. Eur Heart J.

[32] Fisher JA, Puett RC, Laden F (2019). Case-crossover analysis of short-term particulate matter exposures and stroke in the health professionals follow-up study. Environ Int.

[33] Mechtouff L, Canoui-Poitrine F, Schott AM (2012). Lack of association between air pollutant exposure and short-term risk of ischaemic stroke in Lyon, France. Int J Stroke.

[34] Sun S, Stewart JD, Eliot MN (2019). Short-term exposure to air pollution and incidence of stroke in the Women's Health Initiative. Environ Int.

[35] Butland BK, Atkinson RW, Crichton S (2017). Air pollution and the incidence of ischaemic and haemorrhagic stroke in the South London Stroke Register: a case-cross-over analysis. J Epidemiol Community Health.

[36] Lin H, Tao J, Du Y (2016). Differentiating the effects of characteristics of PM pollution on mortality from ischemic and hemorrhagic strokes. Int J Hyg Environ Health.

[37] O'Donnell MJ, Fang J, Mittleman MA, Kapral MK, Wellenius GA (2011). Fine particulate air pollution (PM2.5) and the risk of acute ischemic stroke. Epidemiology.

[38] Villeneuve PJ, Johnson JY, Pasichnyk D, Lowes J, Kirkland S, Rowe BH (2012). Short-term effects of ambient air pollution on stroke: who is most vulnerable?. Sci Total Environ.

[39] (2020). EPA Retains Air Quality Standards for Particle Pollution (Particulate Matter): Fact Sheet. The National Ambient Air Quality Standards for Particulate Matter.

[40] Yuan S, Wang J, Jiang Q (2019). Long-term exposure to PM(2.5) and stroke: a systematic review and meta-analysis of cohort studies. Environ Res.

[41] Qiu H, Sun S, Tsang H, Wong CM, Lee RS, Schooling CM, Tian L (2017). Fine particulate matter exposure and incidence of stroke: a cohort study in Hong Kong. Neurology.

[42] Kim H, Kim J, Kim S (2017). Cardiovascular effects of long-term exposure to air pollution: a population-based study with 900 845 person-years of follow-up. J Am Heart Assoc.

[43] Puett RC, Hart JE, Suh H, Mittleman M, Laden F (2011). Particulate matter exposures, mortality, and cardiovascular disease in the health professionals follow-up study. Environ Health Perspect.

[44] Cai Y, Hodgson S, Blangiardo M (2018). Road traffic noise, air pollution and incident cardiovascular disease: a joint analysis of the HUNT, EPIC-Oxford and UK Biobank cohorts. Environ Int.

[45] Amini H, Dehlendorff C, Lim YH (2020). Long-term exposure to air pollution and stroke incidence: a Danish Nurse cohort study. Environ Int.

[46] Crichton S, Barratt B, Spiridou A (2016). Associations between exhaust and non-exhaust particulate matter and stroke incidence by stroke subtype in South London. Sci Total Environ.

[47] Huang K, Liang F, Yang X (2019). Long term exposure to ambient fine particulate matter and incidence of stroke: prospective cohort study from the China-PAR project. BMJ.

[48] Maheswaran R, Pearson T, Smeeton NC, Beevers SD, Campbell MJ, Wolfe CD (2010). Impact of outdoor air pollution on survival after stroke: population-based cohort study. Stroke.

[49] Chen G, Wang A, Li S (2019). Long-term exposure to air pollution and survival after ischemic stroke. Stroke.

[50] Qian Y, Yu H, Cai B, Fang B, Wang C (2019). Association between incidence of fatal intracerebral hemorrhagic stroke and fine particulate air pollution. Environ Health Prev Med.

[51] Cai B, Xia T, Qian Y, Lu H, Cai R, Wang C (2020). Association between fine particulate matter and fatal hemorrhagic stroke incidence: a time stratified case-crossover study in Shanghai, China. J Occup Environ Med.

[52] Ruan Z, Qi J, Yin P (2020). Prolonged life expectancy for those dying of stroke by achieving the daily PM(2.5) targets. Glob Chall.

[53] Li J, Zhang X, Yin P, Wang L, Zhou M (2020). Ambient fine particulate matter pollution and years of life lost from cardiovascular diseases in 48 large Chinese cities: association, effect modification, and additional life gain. Sci Total Environ.

[54] Andersen KK, Olsen TS, Dehlendorff C, Kammersgaard LP (2009). Hemorrhagic and ischemic strokes compared: stroke severity, mortality, and risk factors. Stroke.

[55] Ahangar AA, Saadat P, Heidari B, Taheri ST, Alijanpour S (2018). Sex difference in types and distribution of risk factors in ischemic and hemorrhagic stroke. Int J Stroke.

[56] Zhang Y, Tuomilehto J, Jousilahti P, Wang Y, Antikainen R, Hu G (2011). Lifestyle factors on the risks of ischemic and hemorrhagic stroke. Arch Intern Med.

[57] Wang HK, Huang CY, Sun YT (2020). Smoking paradox in stroke survivors? Uncovering the truth by interpreting 2 sets of data. Stroke.

[58] Price AJ, Wright FL, Green J (2018). Differences in risk factors for 3 types of stroke: UK prospective study and meta-analyses. Neurology.

[59] Yang BY, Fan S, Thiering E, Seissler J, Nowak D, Dong GH, Heinrich J (2020). Ambient air pollution and diabetes: a systematic review and meta-analysis. Environ Res.

[60] Connor M, Lamorie-Foote K, Liu Q (2021). Nanoparticulate matter exposure results in white matter damage and an inflammatory microglial response in an experimental murine model. PLoS One.

[61] Babadjouni R, Patel A, Liu Q (2018). Nanoparticulate matter exposure results in neuroinflammatory changes in the corpus callosum. PLoS One.

[62] Shkirkova K, Lamorie-Foote K, Connor M (2020). Effects of ambient particulate matter on vascular tissue: a review. J Toxicol Environ Health B Crit Rev.

[63] Calderón-Garcidueñas L, Villarreal-Calderon R, Valencia-Salazar G (2008). Systemic inflammation, endothelial dysfunction, and activation in clinically healthy children exposed to air pollutants. Inhal Toxicol.

[64] Brook RD, Rajagopalan S, Pope CA 3rd (2010). Particulate matter air pollution and cardiovascular disease: an update to the scientific statement from the American Heart Association. Circulation.

[65] Libby P, Buring JE, Badimon L (2019). Atherosclerosis. Nat Rev Dis Primers.

[66] Brook RD, Rajagopalan S (2021). Getting sympathetic about air pollution exposure. J Am Heart Assoc.

[67] Lucking AJ, Lundback M, Mills NL (2008). Diesel exhaust inhalation increases thrombus formation in man. Eur Heart J.

[68] Lu J, Lu Y, Yang H (2019). Characteristics of high cardiovascular risk in 1.7 million Chinese adults. Ann Intern Med.

[69] (2017). Smoking prevalence and attributable disease burden in 195 countries and territories, 1990-2015: a systematic analysis from the Global Burden of Disease Study 2015. Lancet.

[70] Shumake KL, Sacks JD, Lee JS, Johns DO (2013). Susceptibility of older adults to health effects induced by ambient air pollutants regulated by the European Union and the United States. Aging Clin Exp Res.

[71] O'Neill MS, Veves A, Sarnat JA (2007). Air pollution and inflammation in type 2 diabetes: a mechanism for susceptibility. Occup Environ Med.

[72] Song JW, Chung KC (2010). Observational studies: cohort and case-control studies. Plast Reconstr Surg.

